# 4300 Evaluation and structure of the pilot funding program at the University of North Carolina CTSA Hub (NC TraCS)

**DOI:** 10.1017/cts.2020.235

**Published:** 2020-07-29

**Authors:** Kalene Morozumi, Tanha Patel, Tim Carey, John B Buse, Andrea Carnegie, Giselle Corbie-Smith, Gaurav Dave, Mary Beth Cassely, Paul Kerr

**Affiliations:** 1 University of North Carolina School of Medicine; 2 NC TraCS

## Abstract

OBJECTIVES/GOALS: The goals of this evaluation were 1) to describe the pilot grant application cycle and processes at NC TraCS, 2) to illustrate the impact of pilot grants on extramural grant funding, and 3) to provide a framework for other institutions to utilize for the evaluation of pilot grant programs. METHODS/STUDY POPULATION: From 2009-2019 the NC TraCS pilot program funded 925 projects, varying from $2,000 to $100,000. Pilot grants are available to any researcher affiliated with the university as well as partner institutions and community stakeholders. For this evaluation we analyzed data on pilot applicants (demographics, type of pilot, funding status, resubmissions, etc.) and outcomes (extramural funding, publications, etc.) yielded from funded pilots. In addition to summary statistics, we also calculated return on investment (ROI) for the program as a whole and by specific grant type. We will use bibliometric network analysis to assess productivity, citation impact, and scope of collaboration. RESULTS/ANTICIPATED RESULTS: There have been 2,777 submitted proposals with an acceptance rate of 33.3%. Unfunded proposals can resubmit, 61.8% of resubmitted applications are successfully funded, and 29.6% of funded applications are resubmissions. The $2,000 awards accounted for 43.4% of all grants awarded but only accounted for 6.4% of all pilot funds awarded. Success of proposals was proportional to the number of applications from each academic unit. 60.8% of funded applicants were affiliated with the School of Medicine and account for 65.3% of all funding awarded from 2009-2019. Additionally, we plan on analyzing return on investment rates to illustrate the impact of pilot awards on future research funding. DISCUSSION/SIGNIFICANCE OF IMPACT: Pilot grants can lead to subsequent extramural grants, publications, and successful translation of research into practice. This evaluation will assist our institution in understanding the impact of pilot grants and will provide a road map for other institutions evaluating their own programs.

